# Unmasking the “targetless” illusion: branched clonal evolution of *TERT* and *KIT* in acral melanoma revealed by sequential multi-site biopsies- a case report

**DOI:** 10.3389/fimmu.2026.1810921

**Published:** 2026-05-20

**Authors:** Yuqiao Fu, Peng Li, Ke Li, Lei Jiang, Yingzi Liang, Lipei Shao, Ya Zhang, Chunlei Ge, Zhaoqi Zhang, Ying Wang, Ruilei Li

**Affiliations:** 1 Cancer Biotherapy Center & Cancer Research Institute, The Third Affiliated Hospital of Kunming Medical University, Yunnan Cancer Hospital, Peking University Cancer Hospital Yunnan, Kunming, China; 2 Science and Technology Achievement Transformation Center, Kunming Medical University, Kunming, China; 3 Department of Cell Biology, Nanjing Medical University, Nanjing, China; 4 Department of Radiology, The Third Affiliated Hospital of Kunming Medical University, Yunnan Cancer Hospital, Peking University Cancer Hospital Yunnan, Kunming, China

**Keywords:** acral melanoma, branched clonal evolution, *KIT* p.Ala829Pro, spatiotemporal heterogeneity, systemic therapeutic pressure, *TERT* promoter mutation

## Abstract

Tumor clonal evolution and spatiotemporal heterogeneity are key drivers of therapeutic resistance in melanoma, often driven by selection pressures generated by systemic therapy. Genetic testing plays a key role in guiding targeted therapies, but a single biopsy may not fully reflect the mutational landscape. This paper reports a case of metastatic acral melanoma that demonstrated dynamic branching clonal evolution under systemic therapeutic stress. The patient first underwent genetic testing in 2022. A 168-gene panel performed on a newly developed inguinal lymph node metastasis revealed only a *TERT* promoter mutation (c.-124C>T), with no *KIT* mutation detected. Disease progression despite multiple rounds of cytotoxic chemotherapy, antiangiogenic therapy, and immunotherapy. A 10-gene panel testing on the newly developed abdominal skin metastasis in 2024 revealed a rare *KIT* exon 18 mutation (p.Ala829Pro). However, the panel did not include the *TERT* gene. Then our retrospective single-gene testing of this abdominal skin metastasis confirmed the presence of concurrent *TERT* mutations. Retrospective testing of the primary acral lesion from 2019 showed that *TERT* and *KIT* mutations coexisted in a subclonal pattern. The variant allele frequency of *KIT* in the advanced cutaneous metastasis increased significantly, suggesting the possible occurrence allelic imbalance at the *KIT* gene locus. This case demonstrates that the *KIT*-mutant subclone in the advanced cutaneous metastasis was not newly acquired, but rather a pre-existing cell population that was selected for through a stringent therapeutic bottleneck. The study also showed the inherent resistance of the *KIT* p.Ala829Pro mutation to imatinib, explaining the failure of subsequent treatment. This study highlights the significant spatial and temporal heterogeneity of acral melanoma and underscores the critical importance of serial biopsies in accurately capturing clonal dynamics and guiding precision oncology therapy.

## Introduction

Melanoma is a highly malignant tumor that originates from melanocytes and can arise in the skin, mucosa, uveal tract, leptomeninges, and other anatomical sites. It is characterized by a tendency to metastasize early and a highly aggressive nature (1, 2). Although melanoma accounts for only about 4% of all skin malignancies, it is responsible for nearly 80% of skin cancer-related deaths (3). Advances in targeted therapies for specific genetic alternations as well as immune checkpoint inhibitors have significantly improved clinical outcomes for a subset of patients. However, tumor heterogeneity and clonal evolution often lead to treatment failure and disease recurrence. Studies have demonstrated genetic heterogeneity between primary and metastatic tumors within the same melanoma patient. In a multi-platform analysis of primary tumors and multiple metastases from 99 melanoma patients, Chang et al. reported that approximately 18% of patients showed discordance in *BRAF*, *NRAS*, or *TERT* mutation status across different tumor lesions (4). A systematic review indicated pooled concordance rates of 88.8% for *BRAF* and 97.2% for *NRAS* mutation status between primary tumors and metastatic lesions, though variability across individual studies exists based on detection methods, patient gender, and metastatic sites (5).

This report documents the branching clonal evolution of the tumor genome of a patient with metastatic acral melanoma over a 5-year time span and after multiple cycles of chemotherapy, anti-angiogenic therapy, and immunotherapy, showing a high degree of spatiotemporal heterogeneity.

## Case presentation

A 50-year-old male presented with a black lesion on the sole of his left foot that had been present for more than a year and had gradually increased in size. The patient underwent lesion excision at a local hospital in November 2019, and postoperative pathology confirmed malignant melanoma. The patient was then referred to our hospital for further examination and treatment. Our histopathology consultation determined the lesion to be a melanoma without ulceration, with a Breslow thickness of 1.3 mm, a Clark classification of II-III, a mitotic rate of 3/mm², and no tumor cells at the surgical margins or at the base. PET/CT imaging showed no metastases. The tumor was staged as T2aN0M0(stage IB).

In December 2019, physical examination noted a dark spot remained adjacent to the surgical site, but the patient declined re-excision. Comprehensive examinations revealed no significant tumor residue or metastasis. Subsequently, high-dose interferon therapy was initiated on December 6, 2019(18 million IU, 5 days/week for 4 weeks, followed by 9 million IU, 3 days/week for 11 months). Regular follow-up showed stable disease until June 2022.

**Figure 1:**
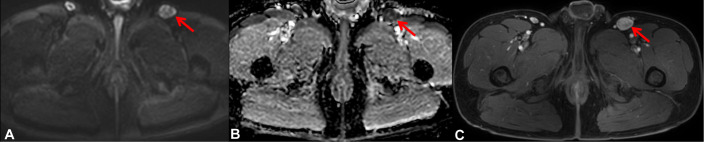
Magnetic resonance imaging on August 13, 2022 showed multiple enlarged lymph nodes were seen adjacent to the left external iliac vessels and in the left inguinal region, which were considered metastatic, and the large one was located in the left inguinal region, which was about 2.3 cm × 1.7 cm in size. **(A)** Axial diffusion-weighted imaging (DWI). **(B)** Axial apparent diffusion coefficient (ADC) map. **(C)** Axial fat-suppressed, contrast-enhanced T1-weighted (T1-fs+C) image.

In August 2022, imaging revealed new left iliac paravascular and inguinal lymph node metastases (**Figure 1**). On August 20, 2022, the patient underwent left inguinal lymph node dissection along with the excision of the aforementioned persistent dark spot adjacent to the original surgical site. Postoperative pathology confirmed metastasis in 11 of 23 lymph nodes; the excised acral dark lesion was confirmed to be an atypical junctional nevus, ruling out a second primary melanoma. Staging: T2aN3bM1a, stage IV. Genetic testing of the left inguinal lymph node metastasis using a 168-gene panel (based on next-generation sequencing (NGS) with target region capture on the Illumina platform) detected a *TERT* c.-124C>T mutation (variant allele frequency [VAF], 33.33%), without *KIT*, *BRAF*, or *NRAS* mutations. On December 10, 2022, January 10, 2023, and February 14, 2023, the patient received three cycles of temozolomide chemotherapy, respectively. Follow-up examinations showed stable disease.

**Figure 2:**
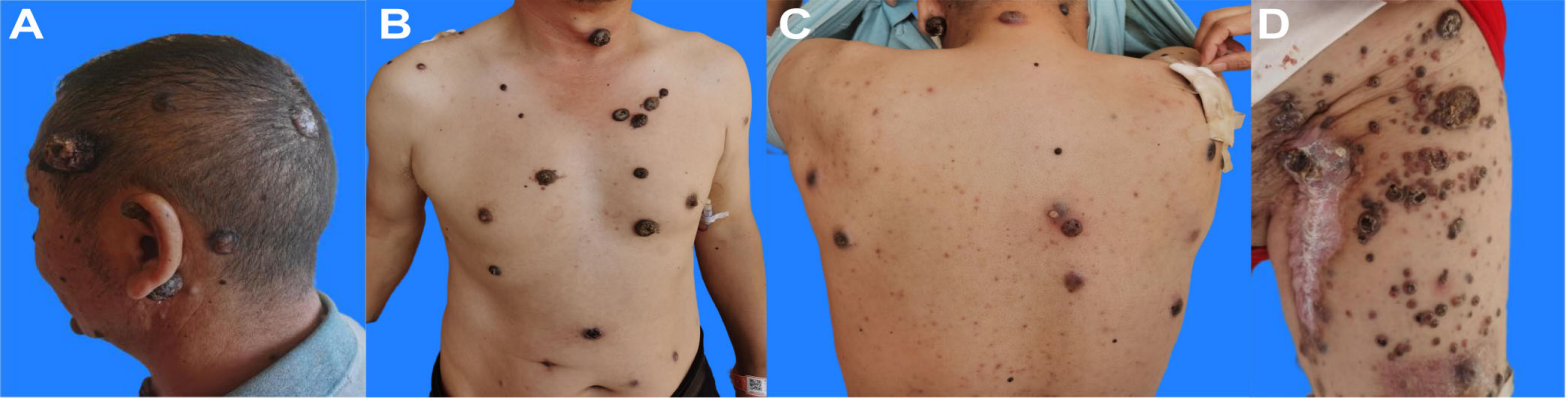
Cutaneous metastases in the patient with melanoma. The photographs show multiple, variably sized and shaped, black to dark-brown nodules on the **(A)** scalp and retroauricular area; **(B)** anterior neck, chest, and abdomen; **(C)** posterior neck and back; and **(D)** left upper thigh and inguinal region.

**Figure 3:**
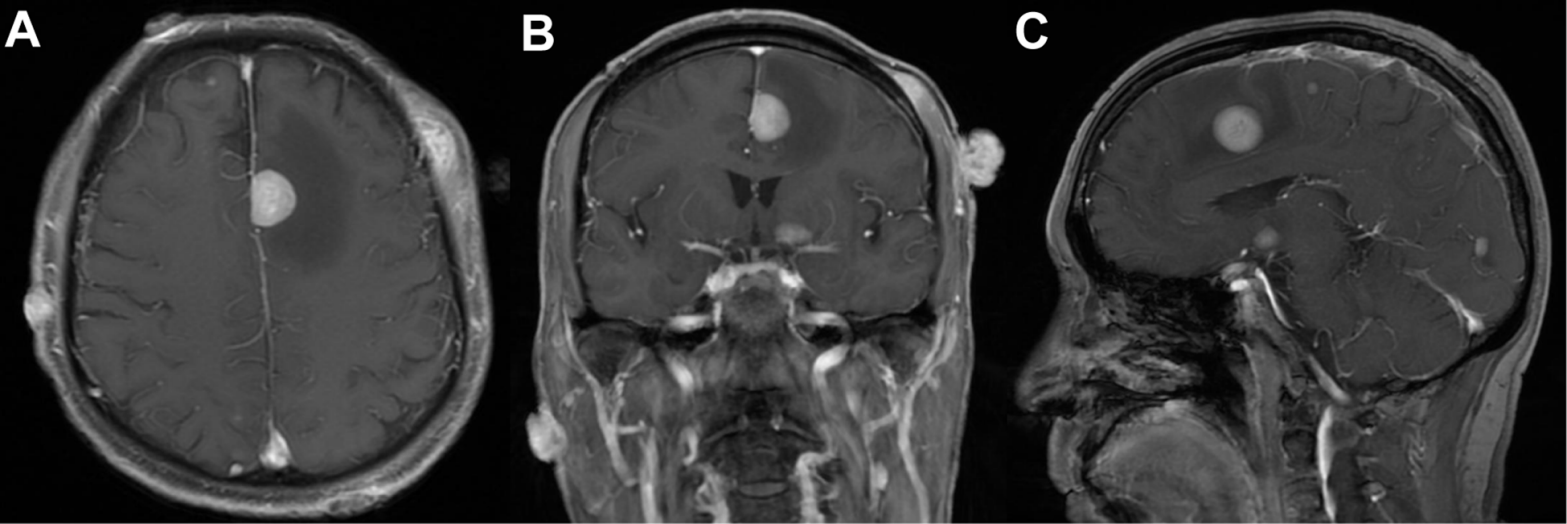
Magnetic resonance imaging of the brain on August 24, 2023 demonstrate numerous heterogeneously enhancing intracranial lesions, suggestive of brain metastases. **(A)** Axial, **(B)** coronal, and **(C)**​ sagittal fat-suppressed, contrast-enhanced T1-weighted images.

**Figure 4:**
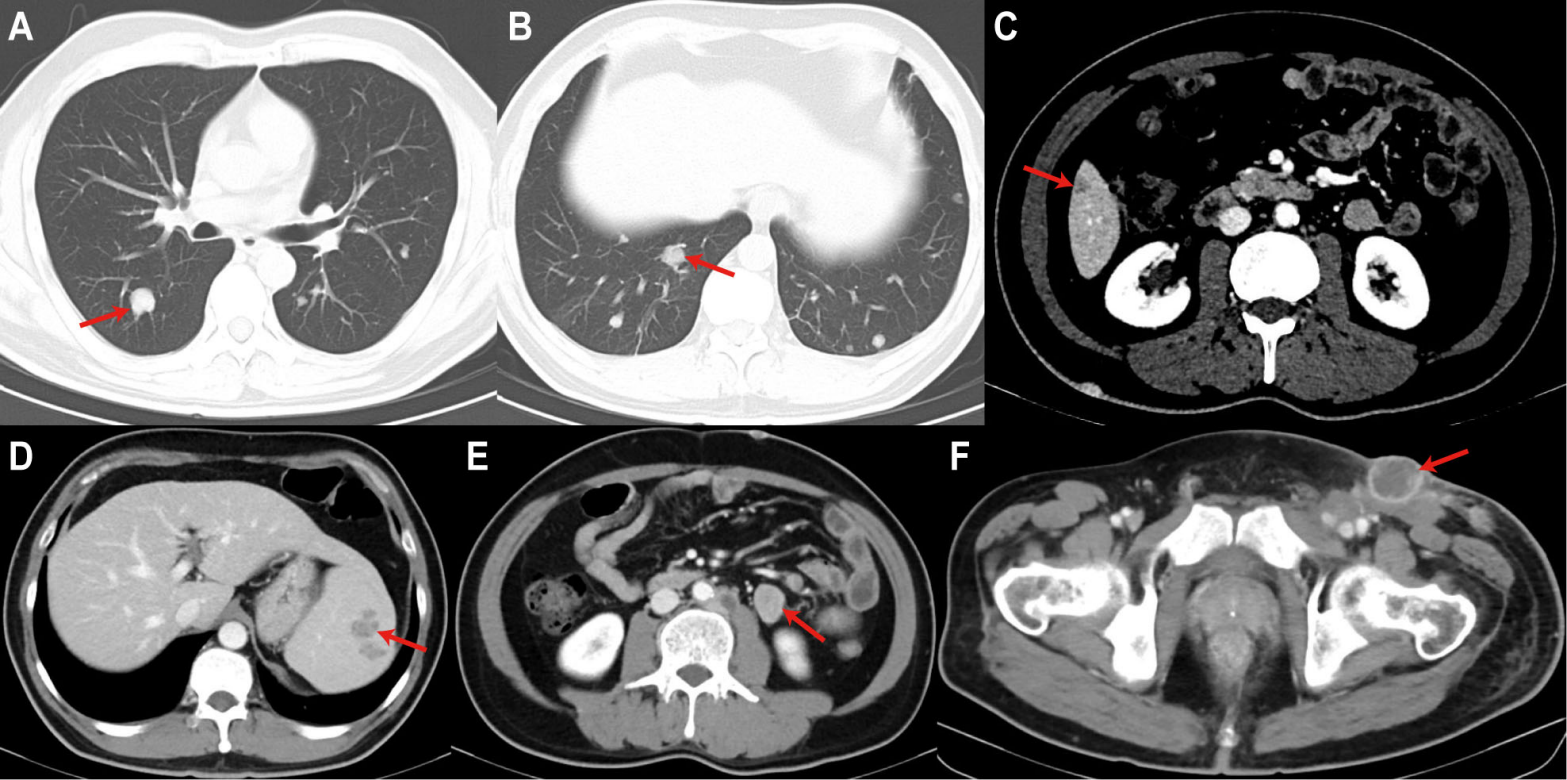
Enhanced CT scan on August 24, 2023, revealed multiple systemic organ metastases. **(A, B)** Multiple nodules in both lungs; **(C)** A subcapsular nodule in the inferior segment of the right anterior hepatic lobe; **(D)** Multiple nodules in the spleen; **(E)** A nodule in the left adrenal gland; **(F)** Enlarged lymph nodes in the left inguinal region. All of the above findings are considered metastatic.

In March 2023, multiple black nodules appeared on the face. Ultrasound findings were suspicious for metastatic melanoma. The patient subsequently underwent excision of the facial masses. Postoperative pathology confirmed melanoma. Between March and August 2023, multiple black masses appeared on the patient’s body and progressively increased in number and size, but the patient received no treatment (**Figure 2**). On August 22, 2023, follow-up imaging revealed rapid systemic progression of the disease, demonstrating metastases to the brain (**Figure 3**), lungs, liver, spleen, left adrenal gland, inguinal lymph nodes, as well as widespread cutaneous and subcutaneous involvement (**Figure 4**). The staging was updated to T2aN3cM1d, stage IV. The patient received two cycles of nab-paclitaxel plus carboplatin chemotherapy, concurrently with palliative whole-brain radiotherapy (30 Gy in 10 fractions). Imaging evaluation in October 2023 demonstrated stable disease. And bevacizumab was added during the third and fourth treatment cycles, administered on October 24 and November 21, 2023, respectively. However, disease progression was observed on follow-up assessment in December 2023. Consequently, the PD−1 inhibitor toripalimab was combined in the fifth and sixth cycles, which were given on December 22, 2023, and January 18, 2024, respectively. In February 2024, the patient developed a new abdominal skin lesion. So we performed a second genetic test on the lesion using a melanoma-specific 10-gene panel that covers *BRAF*, *CTNNB1*, *GNA11*, *GNAQ*, *KIT*, *KRAS*, *MAP2K1*, *NRAS*, *PDGFRA*, and *TP53*. The analysis identified a *KIT* exon 18 mutation (c.2485G>C, p.Ala829Pro) with a VAF of 32.73%. After departmental discussion, we gave the patient 1 cycle of the combination regimen of temozolomide, imatinib and toripalimab. The patient subsequently developed aphasia, cognitive impairment, and seizures, indicating poor treatment tolerance. As a result, the regimen was deescalated to PD-1 inhibitor monotherapy. Despite these treatments, the patient’s condition continued to deteriorate and he eventually passed away on May 11, 2024. To better understand the genetic evolution of the tumor in this patient, we performed two retrospective tests after the patient’s departure: 1) since the melanoma-specific 10-gene panel did not contain the *TERT* gene, we performed complementary *TERT* single-gene testing of the 2024 abdominal skin metastasis, which showed the *TERT* c.-124C>T mutation (VAF 33.8%), confirming the coexistence of *TERT* and *KIT* mutations within the same lesion; 2) We supplemented genetic testing of the 2019 acral primary lesion with a 19-gene panel, which confirmed that the *TERT* c.-124C>T mutation (VAF 10.70%) and the *KIT* p.Ala829Pro (VAF 8.50%) mutation coexisted as subclones from the early stage of the tumor (**Supplementary Figure 1**). Comprehensive sequencing metrics across all biopsies, including average depth, tumor purity, and variant allele frequencies, are detailed in **Supplementary Table 1**.

## Discussion

### Tumor spatiotemporal heterogeneity and branching evolution

There is a certain tumor heterogeneity in melanoma, which includes both temporal and spatial dimensions.Within the same patient, differences in genetic characteristics may exist between the primary tumor and metastatic lesions, as well as before and after treatment. Harbst et al. reported that 50% of melanoma patients showed phenotypic differences among metastases, and 82% showed genetic inconsistency, with one or more metastases carrying “private” somatic mutations unique to individual tumors (6). This case demonstrates the branching clonal evolution of acral melanoma by longitudinal multisite biopsy. To assess the clonal structure, we used the cancer cell fraction (CCF) to measure the proportion of cancer cells carrying a specific somatic mutation in the tumor cell population (7). In most somatic mutation analyses, assuming that the human genome is diploid and that early-occurring point mutations in oncogenic driver genes are usually heterozygous, the theoretical formula for CCF is as follows (8):


$$\text{CCF}=\frac{2\text{VAF}}{\rho }$$

where ρ is the tumor purity. Tumor purity was histologically evaluated by a pathologist through hematoxylin and eosin (H&E) staining of the sample prior to DNA extraction. Based on this formula, if a heterozygous mutation is present in all tumor cells, the theoretical maximum VAF should be strictly equal to half of the tumor purity. When the observed VAF is significantly lower than this theoretical threshold, it suggests that the mutation exists only as a subclonal mutation in some tumor cells. Conversely, if the observed VAF is significantly higher than this threshold, it suggests the presence of secondary genomic abnormalities. These abnormalities usually include absolute mutant allele amplification or loss of heterozygosity (LOH), especially copy-neutral LOH (CN-LOH), in which the wild-type allele is cleared while the mutant allele is amplified, thus maintaining the overall diploid state of the cell (9).

In the acral primary lesion of 2019, the CCFs with *TERT* and *KIT* mutations were approximately 71.3% and 56.6%, respectively. This suggests that the primary tumor is not composed of a single clone but show significant subclonal heterogeneity. Previous studies have demonstrated that in the clonal evolution of melanoma, *TERT* mutations are usually the trunk event in the early stage of the tumor. Wang et al. demonstrated that *TERT* activation (including promoter mutations, amplification, etc.) is an early event in acral melanoma, while MAPK pathway mutations (e.g. *BRAF*, *NRAS*, *KIT*) emerge later, fostering clonal diversity (10). Thus, at least two cell populations exist within this primary tumor: one carrying only *TERT* mutations and the other carrying both *TERT* and *KIT* double mutations.

In 2022, we performed genetic testing on the inguinal lymph node metastasis with a 168-gene panel. The results showed only a *TERT* promoter mutation (VAF 33.33%). According to this formula, the theoretical maximum VAF for a heterozygous mutation should be 20% at 40% tumor purity, but the observed VAF for *TERT* mutations significantly exceeds this theoretical upper limit. Although there may be differences in the assessment of tumor purity based on H&E staining, this abnormally high frequency still suggests that there may be an allelic imbalance at the *TERT* locus, and because no amplification of the *TERT* gene was detected by this genetic test, this suggests the tumor may have loss of heterozygosity (LOH) at the *TERT* locus. Notably, we did not detect any low abundance mutations in the *KIT* gene, despite the fact that the average sequencing depth of this genetic test was as high as 1610×, which can capture low-frequency mutations down to 3% (11). This suggests spatial heterogeneity of melanoma and its branching clonal evolutionary features. The *KIT* wild-type subclone in the primary focus may have selectively metastasized to the regional lymph node, creating the illusion that no treatable target was identified in the first genetic testing of this patient. However, we also had to consider another possibility, albeit less likely, that a small number of *KIT* mutant subclones may have been present in this inguinal lymph node metastasis, but in quantities that were below the detection limit of the NGS assay panel used. This case demonstrates that biopsies relying on a single spatial site are not representative of the tumor as a whole, and that there is a serious sampling bias. Inter-tumoral heterogeneity may originate from pre-existing intra-tumoral heterogeneity within the primary tumor and is further amplified by the dissemination and cloncal expansion of genetically diverse circulating tumor cells during metastatic spread (12).

The dynamic evolution of the tumor genome is not a simple accumulation of random mutations, but rather the result of treatment-mediated Darwinian selection pressures (13). After two years of treatment with a combination of chemotherapy, anti-angiogenic drugs, and immunotherapy, the *KIT* mutation was again detected in the new abdominal cutaneous metastasis in this patient in 2024. Because a small number of *KIT* mutant subclones were already present in the primary lesion, the recurrence of the *KIT* mutation in this abdominal cutaneous metastasis was not a *de novo* mutation, but rather a transfer of the *KIT* mutant subclone initially present in the primary lesion and selective clonal amplification under the pressure of the prolonged multidrug combination therapy. At 50% tumor purity, the theoretical maximum VAF for *KIT* heterozygous mutations should be 25%, but the observed VAF was as high as 32.73%. Since no absolute *KIT* gene amplification was detected by this genetic test, even though there may be a difference in the assessment of tumor purity based on H&E staining, this value still suggests that this lesion may have an allelic imbalance or partial LOH at the *KIT* locus.

### *TERT* promoter alterations in acral melanoma: phylogenetic truncal driver status and prognostic value

Although the *TERT* c.-124C>T mutation exists as a subclone in the primary lesion, it was prevalent between the primary lesion and subsequent metastases over a time span of nearly five years, suggesting its foundational position in the phylogenetic trunk of clonal evolution.

Every mitotic division of human somatic cells is accompanied by a shortening of the telomeres at the ends of the chromosome. Once the telomeres shorten to a certain length, the cell initiates the senescence or apoptosis program. The “Hayflick limit” can limit the proliferative potential of primary cells, but it can be breached by overexpression of *TERT* or *TERT* promoter mutations, giving cells the ability to proliferate indefinitely (14, 15). *TERT* promoter mutations drive tumorigenesis through a two-step mechanism. In the early tumorigenesis, *TERT* promoter mutations upregulate telomerase activity by creating new ETS transcription factor binding sites. Although this up-regulation is not sufficient to maintain telomere length, it delays cellular senescence, giving it additional proliferative capacity in the face of persistent telomere shortening. As telomeres shorten to a critical length, genomic instability increases, which in turn selectively continues to upregulate telomerase activity, allowing cells to continue proliferating (15). *TERT* promoter mutations, specifically the c.-124C>T and c.-146C>T variants, are highly prevalent in melanoma (16). However, statistical analysis showed that *TERT* promoter mutations occurred with lower frequency in acral or mucosal melanoma than in the cutaneous type (17). Studies have shown that melanoma harboring *TERT* mutation (especially c.-124C>T) tends to exhibit thicker Breslow thickness, higher incidence of ulceration, and greater susceptibility to neurologic invasion (18). Theoretically, synergistic oncogenic effects can occur when *TERT* promoter mutations coexist with MAPK pathway driver mutations (e.g., *KIT*). The MAPK pathway provides sustained mitogenic signaling to drive rapid cell proliferation; whereas *TERT* promoter mutations resolve telomere crisis through a stepwise adaptation mechanism. The patient developed extensive systemic metastases at an advanced stage, suggesting a potentially high risk for this molecular combination, but its clear clinical prognostic significance needs to be confirmed by additional studies.

### Rare *KIT* p.Ala829Pro mutation and intrinsic resistance

*KIT* is a proto-oncogene encoding a receptor tyrosine kinase that drives cell growth and proliferation by activating the MAPK and PI3K-AKT pathways (19). *KIT* mutations can occur in multiple hotspot regions, but different mutation sites have different sensitivities to *KIT* inhibitors such as imatinib. *KIT* exon 11 and 13 mutations are highly sensitive to imatinib, while exon 17 mutations are almost or completely insensitive (20). The *Kit* p.Ala829Pro (A829P) mutation is located within the exon 18 kinase activation loop and is extremely rare in melanoma. In the vitro pharmacological study showed that activation loop mutations not only lead to sustained activation of the ligand-independent kinase, but also shift the kinase conformation towards the active state. Since imatinib is a type II inhibitor that must bind to the inactive conformation, the change in kinase conformation triggered by the activating loop mutation leads to a strong steric clash between the drug and the DFG motif of the kinase (especially Phe811), resulting in primary resistance (21). Todd et al. reported that melanoma with *KIT* A829P mutation is completely resistant to imatinib and sunitinib, but they may be partially sensitive to second-generation TKIs such as nilotinib and dasatinib (22). In this case, the patient developed aphasia, cognitive impairment, and seizures after receiving only one cycle of a combination of temozolomide, imatinib, and toripalimab. This rapid deterioration may be the result of a combination of complex factors. Imatinib does not easily penetrate the blood-brain barrier (23), and the *KIT* A829P mutation produces primary resistance to imatinib, resulting in widespread brain metastases that cannot be effectively controlled even with targeted therapies, which leads to numerous neurological symptoms. At the same time, the patient was already in the terminal stage of the tumor, with a poor baseline state, and was no longer able to tolerate the toxic effects of this intensive triple therapy overlay.

### Limitation

We acknowledge that in this case, we used different target panels (168 genes vs. 10 genes) to perform bulk NGS sequencing of the patient’s different metastases at different time points during the course of the consultation, which may have resulted in differences in sequencing depth and sensitivity. We did not detect any low-frequency *KIT* mutations in the 2022 lymph node metastasis using a 168-gene panel (average sequencing depth 1610×). Although we are more likely to consider the association with tumor branching clonal evolution, we cannot exclude the possibility that there are *KIT* mutant subclones that also metastasize simultaneously to the inguinal lymph node, but are unable to outcompete the *KIT* wild-type clones in the specific lymphatic microenvironment, resulting in a very low percentage of *KIT* mutations. Even at an average sequencing depth of 1610× (which can capture as low as 3% of low-frequency mutations), very low VAF can fall below the detection limit, resulting in false-negative result. However, based on the selectivity of the current clinical panel and the average sequencing depth, we were unable to retest the inguinal lymph node metastasis in 2022 using the melanoma-specific 10-gene panel. Although we inferred potential allelic imbalances based on differences between tumor purity and VAF, these estimates are inherently semiquantitative. Bulk NGS can only provide the average signal of cell populations, and to ultimately determine the genomic structure within a complex lesion, we will still need to use single-cell DNA sequencing for in-depth analysis in the future.

## Conclusion

This case documents the branching clonal evolution of metastatic acral melanoma under systemic therapeutic stress. If we rely only on single biopsy samples, we will be at risk of significant sampling bias, which may mask the true spatio-temporal heterogeneity of the tumor. In addition, this case shows that the *KIT* p.Ala829Pro mutation, which is rarely present in melanoma, is primary resistant to imatinib. It is critical that we perform sequential multiregional biopsies and longitudinal molecular monitoring during personalized and precise treatment of acral melanoma.

## Data Availability

The original contributions presented in the study are included in the article/**Supplementary Material**. Further inquiries can be directed to the corresponding authors.
