# Supplementary material for: Unmasking the “targetless” illusion: branched clonal evolution of TERT and KIT in acral melanoma revealed by sequential multi-site biopsies- a case report
Source: Front Immunol. 2026 May 20;17:1810921. doi: 10.3389/fimmu.2026.1810921 (PMC13230091; doi:10.3389/fimmu.2026.1810921)
Supplement: Supplementary file 2 [file Image1.pdf]

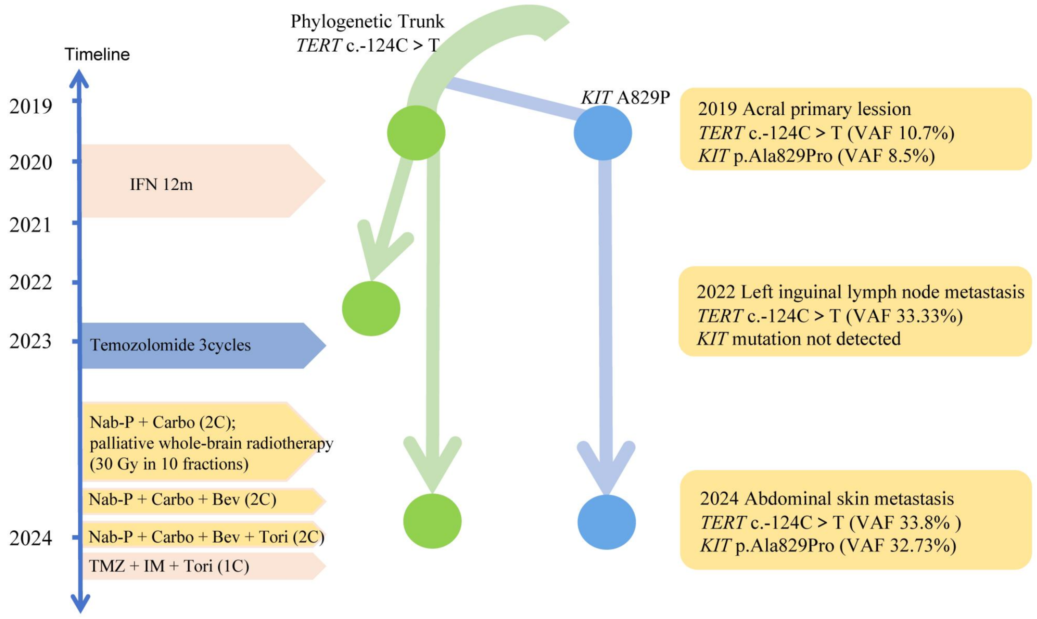

Supplementary Figure 1. Schematic representation of the branching clonal evolution of *TERT* and *KIT* mutations across sequential multi-site biopsies under systemic therapeutic pressures.
